# Supplementary material for: WordCommentsAnalyzer: A windows software tool for qualitative research
Source: F1000Res. 2018 Sep 4;7:536. Originally published 2018 May 3. [Version 2] doi: 10.12688/f1000research.14819.2 (PMC6137411; doi:10.12688/f1000research.14819.2)
Supplement: Supplementary file 3 [file f1000research-7-17614-s0002.tgz › b2a79496-4ada-47c4-9682-a99ce867836d.zip!/Eight Iranian Health Journals/Salmand_Iranian Journal of Ageing _29.docx]

© Salmand_Iranian Journal of Ageing . This work is licensed under CC BY-NC 4.0 (https://creativecommons.org/licenses/by-nc/4.0/).

Volume & Issue: دوره 6 - شماره 1

Title: تأثیر تمرین‌های ورزشی هوازی بر کیفیت زندگی سالمندان مقیم سرای سالمندان شهرستان اهواز

http://salmandj.uswr.ac.ir/article-1-376-fa.html

اهداف: این مطالعه با هدف بررسی تأثیر تمرینات هوازی بر کیفیت زندگی سالمندان انجام شد. مواد و روش‌ها: در این مطالعه نیمه‌تجربی که در مراکز سالمندان شهرستان اهواز در سال 1389 انجام شد، 50 سالمند (زن ومرد )، دارای توانایی انجام فعالیت ورزشی، انجام فعالیت‌های معمول و روزانه بدون وابستگی به دیگران، فاقد بیماری‌های سیستمیک و محدودکننده، به روش در دسترس انتخاب و به طور تصادفی به دو گروه آزمون و شاهد تقسیم شدند. در گروه آزمون، برنامه ورزشی به مدت 8 هفته (هفته‌ای 3 بار ) اجرا شد. سطح کیفیت زندگی هر دو گروه، قبل از مداخله به وسیله پرسش‌نامه 36-SF ارزیابی شد. از آزمون T- زوجی و T- مستقل، من‌ویتنی و کای دو جهت تجزیه و تحلیل آماری استفاده شد. یافته‌ها: با به‌کارگیری برنامه ورزشی منظم و مدون، سطح کیفیت زندگی سالمندان به طور معنی‌داری نسبت به قبل از اجرای برنامه ورزشی افزایش یافت (0.05p<). در حالی که در همان مدت زمان، گروهی که برنامه ورزشی را تمرین نمی‌کردند تغییر معنی‌داری در ابعاد کیفیت زندگی ایجاد نشده است. نتیجه‌گیری: با توجه به یافته‌های حاصل از این پژوهش می‌توان نتیجه گرفت با به‌کارگیری برنامه ورزشی منظم، مداوم و دارای پذیرش خوب از سوی این گروه سنی، می‌توان سطح کیفیت زندگی را در ابعاد مختلف افزایش داد و راه را برای سالم پیر شدن و برخورداری از سلامتی بیشتر در این دوره از زندگی هموار نمود.

Title: تأثیر تمرینات ویبریشن و مکمل تغذیه‌ای کراتین بر عملکرد پایین تنه و تعادل مردان سالمند

http://salmandj.uswr.ac.ir/article-1-375-fa.html

اهداف: هدف از انجام پژوهش تعیین تأثیر تمرین ویبریشن بر عملکرد (WBVT) و مکمل تغذیه‌ای کراتین (Cr) بر عملکرد پایین‌تنه و تعادل مردان سالمند بود. مواد و روش‌ها: پژوهش از نوع کاربردی و نیمه تجربی است. جامعه آماری پژوهش مردان سالمند بالای 60 سال، عضو در مرکز روزانه سرای سالمندان بودند، که بدین منظور 22 سالمند مرد واجد شرایط (سن 6/84±70/36، وزن 13/63±69/67 کیلوگرم، قد 6/98±166 سانتی‌متر و شاخص توده بدن (BMI) (3/54±24/95) انتخاب و به صورت تصادفی به سه گروه تمرین ویبریشن و مکمل کراتین (WBVT+Cr) 7 نفر، تمرین ویبریشن و دارونما (WBVT+P) 7 نفر و کنترل (C) 8 نفر تقسیم شدند. گروه WBVT+Cr تمرینات را به مدت 10 روز و بر اساس اصل اضافه بار با فرکانس 35-30 هرتز و دامنه 5 میلی‌متر انجام دادند و 20 گرم کراتین در روز در 5 روز اول و در ادامه 5 گرم کراتین به صورت روزانه مصرف کردند. گروه WBVT+P علاوه بر تمرین ویبریشن به جای کراتین از دکستوز استفاده کردند. تعادل ایستا و پویا با آزمون‌های استورک و بلندشدن و راه‌رفتن و عملکرد حرکتی پایین‌تنه با آزمون‌های زمان راه رفتن 30 متر، بلندشدن و نشستن و راه رفتن 6 متر (تاندم گیت) ارزیابی شدند. برای بررسی تفاوت بین گروه‌ها از آزمون تحلیل واریانس یک‌راهه و برای بررسی تفاوت پیش آزمون و پس آزمون از آزمون t وابسته استفاده شد. یافته‌ها: نتایج آزمون t وابسته نشان داد که تعادل پویا، 30 متر راه‌رفتن و تاندم گیت در دو گروه تجربی بهبود یافته است. اما نتایج بین گروهی نشان داد که در رابطه با تعادل ایستا (0/514P=)، تعادل پویا  (0/153P=) و عملکرد پایین‌تنه که با آزمون‌های 30 متر راه‌رفتن  (0/339P=)، نشستن و برخاستن  (0/578P=) و تاندم گیت  (0/151P=) اندازه‌گیری شده بود، تفاوت معنی‌داری وجود ندارد. نتیجه‌گیری: بر اساس نتایج پژوهش، به نظر می‌رسد که یک دوره 10 روزه تمرینات ویبریشن با مکمل تغذیه‌ای کراتین می‌تواند باعث بهبود برخی از عوامل آمادگی حرکتی در مردان سالمند شود.

Title: تأثیر مشارکت در گروه‌های حمایتگر بر سلامت معنوی و فشار مراقبتی در «مراقبان عضو خانواده» سالمندان مبتلا به آلزایمر

http://salmandj.uswr.ac.ir/article-1-374-fa.html

اهداف: نظر به اهمیت توانمندسازی و حمایت مراقبان خانوادگی، این مطالعه به بررسی تأثیر مشارکت فعال مراقبان در گروه‌های حمایتگر بر فشار مراقبتی و بهزیستی معنوی آنان پرداخت. مواد و روش‌ها: در این مطالعه نیمه‌تجربی تک‌گروهه با اندزه گیری مکرر، 32 مراقب خانوادگی عضو انجمن آلزایمر ایران، که حداقل یک سال تجربه مراقبت داشتند در مطالعه وارد شدند و طی 4 ماه در 32 جلسه منظم گروهی، درخصوص بیماری آلزایمر، اصول مدیریت مراقبت از سالمند و خود، حمایت معنوی و روانی از یکدیگر و رویایی و حل مشکلات مراقبت مطرح شد و به تدریج رهبری گروه به اعضای داوطلب سپرده شد. جلسات در سطح جامعه و با مدیریت و برنامه ریزی اعضای گروه‌ها در دو فرهنگسرا و یک مرکز مراقبت روزانه سالمندان بر گزار شد. داده‌ها از طریق پرسش‌نامه‌های سنجش فشار مراقبت و بهزیستی معنوی جمع‌آوری و با آزمون تحلیل واریانس با داده‌های تکراری در سطح معنی‌داری 0/05 تحلیل شد. یافته‌ها: میانگین بهزیستی معنوی طی سه بار اندازه‌گیری به ترتیب 26/029 و 34/029 و 34/471 و برای فشار مراقبتی به ترتیب 40/118، 32/706، 31/265 بود. تحلیل واریانس تفاوت معنی‌داری را در سه بار اندازه‌گیری برای بهزیستی معنوی (0/005P=) و فشار مراقبتی (0/001P≤) نشان داد. نتیجه‌گیری: مشارکت فعال در گروه‌های حمایتگر برای مراقبان، مصداق توانمندسازی آنان برای رویارویی با مشکلات مراقبت از سالمندان مبتلا به آلزایمر است. برنامه‌هایی از این دست، با کاهش فشار مراقبتی و ارتقای سلامت معنوی، منجر به رویایی مؤثرتر مراقب با عوارض منفی مراقبت می شوند. حفظ و ارتقای سلامت مراقبان به استمرار برنامه‌های آموزشی و حمایتی از آنان نیازمند است و این مهم نیز ضرورت تدوین سیاست‌های بهداشتی و حمایتی مؤثر را می‌طلبد.

Title: اثربخشی درمان خاطره‌پردازی انسجامی بر نشانه‌های افسردگی سالمندان

http://salmandj.uswr.ac.ir/article-1-373-fa.html

اهداف: هدف اصلی این پژوهش تعیین اثربخشی درمان خاطره‌پردازی انسجامی بر کاهش نشانه‌های افسردگی سالمندان مقیم مرکز بود. مواد و روش‌ها: طرح پژوهش حاضر از نوع آزمایشی با پیش آزمون و پس آزمون همراه با گروه کنترل بود. جامعه آماری همه سالمندان مقیم مراکز سالمندان شهر اصفهان بودند. انتخاب نمونه‌ها از طریق نمونه‌گیری تصادفی ساده بود. ابتدا پرسش‌نامه افسردگی سالمندان و آزمون معاینه مختصر وضعیت روانی اجرا شد. سپس افرادی که در آزمون افسردگی نمره بالای ده (مبتلا به افسردگی متوسط و شدید) و در آزمون معاینه مختصر وضعیت روانی نمره بالای بیست و یک (از لحاظ شناختی سالم) کسب کردند، مشخص شده و 36 نفر از آن‌ها به‌طور تصادفی انتخاب شدند و به صورت تصادفی به دو گروه 18 نفری آزمایش و کنترل اختصاص یافتند. مداخله به صورت هفتگی در شش جلسه یک ساعت و نیم با موضوعات خاص هر جلسه برگزار شد. تجزیه و تحلیل نتایج با استفاده از آزمون‌های آماری تی برای نمونه‌های مستقل و آزمون خی دو انجام شد. یافته‌ها: میانگین تفاضل پیش آزمون و پس آزمون در گروه آزمایش (2/5385M= و 0/87706SD=) و در گروه کنترل (1M= و 0/93541SD=) به دست آمد. آزمون  t نشان داد که تفاوت دو گروه معنادار بوده و درمان خاطره‌پردازی انسجامی به‌طور معناد‌اری منجر به کاهش نشانه‌های افسردگی سالمندان شد (0/005P<). نتیجه‌گیری: نتایج این تحقیق نشان داد مداخله‌های خاص خاطره‌پردازی می‌تواند مؤثر باشد، و بر اساس نتایج به‌دست آمده، این مداخله را می‌توان در مراکز سالمندان به عنوان درمان کمکی در کنار دارو درمانی استفاده کرد.

Title: عوامل خطر بیماری‌های عروق کرونر در سالمندان مقیم یا غیرمقیم خانه سالمندان

http://salmandj.uswr.ac.ir/article-1-372-fa.html

اهداف: این مطالعه با هدف بررسی عوامل خطر بیماری‌های عروق کرونر در سالمندان مقیم و غیرمقیم در خانه سالمندان انجام شد. مواد و روش‌ها: مطالعه توصیفی - تحلیلی حاضر بر نمونه‌ای متشکل از 382 سالمند مرد و زن بالای 65 سال (191 نفر غیرمقیم و 191 نفر مقیم خانه سالمندان) شهر تهران (1388) انجام شد. نمو نه‌گیری با روش تصادفی - خوشه‌ای انجام شد. از بین خانه‌های سالمندان در مناطق مختلف استان تهران سالمندان مقیم خانه سالمندان انتخاب شدند و با مراجعه منزل به منزل در مناطق همسان سالمندان غیرمقیم انتخاب شدند. از پرسش‌نامه پژوهشگرساخته با مصاحبه حضوری و همچنین اندازه‌گیری قد و وزن (شاخص توده بدن)، فشارخون، قند خون ناشتا و چربی‌های خون استفاده شد. تجزیه و تحلیل داده‌ها با آزمون کای دو و فیشر و از طریق نرم افزار 17 spss در سطح معناداری (0/05P<) انجام شد. یافته‌ها: نتایج نشان داد شیوع عوامل خطرزا در گروه سالمندان غیرمقیم و مقیم خانه سالمندان به ترتیب شامل فشار خون سیستولیک (36/6درصد و 26/2درصد) و دیاستولیک (31/9درصد و 24/1درصد)، کلسترول بالا (36/1درصد و 28/3درصد)،تری‌گلیسرید بالا (42/9درصد و 31/4درصد)،چاقی (22درصد و 12/6درصد)،دیابت (17/3درصد و 12درصد)، عدم تحرک (7/4درصد و 18/8درصد) و نداشتن فعالیت‌های ورزشی (16/2درصد و 56/6درصد)،رویارویی با بیش از 7 عامل استرس‌زا (2/1درصد و 22/5درصد) بود. نتیجه‌گیری: عوامل خطرزای بیماری‌های عروق کرونر در سالمندان شیوع بالایی دارد. همچنین شیوع تمام این عوامل به جز تحرک، فعالیت‌های ورزشی و رویارویی با استرس در سالمندان غیرمقیم نسبت به سالمندان مقیم خانه سالمندان بیشتر بود. لذا با آموزش سالمندان در رابطه با شیوه‌های صحیح سبک زندگی می‌توان از عوامل خطرزای بیماری عروق کرونر و درنتیجه عوارض ناشی از آن پیشگیری کرد.

Title: پاسخ به درمان در سالمندان و معلولان مبتلا به انگل‌های روده‌ای

http://salmandj.uswr.ac.ir/article-1-371-fa.html

اهداف: این مطالعه جهت ارزیابی پاسخ به درمان انگل‌های روده‌ای در سالمندان و معلولان انجام شد. مواد و روش‌ها: این مطالعه توصیفی بر 133 نفر از سالمندان و معلولان مرکز گلاب‌چی کاشان که مبتلا به انگل روده‌ای تشخیص داده شده بودند (1386) انجام شد. درمان افراد آلوده براساس نتیجه آزمایش مدفوع و چسب اسکاچ صورت گرفت. 2 هفته پس ازاتمام درمان مجدداً این آزمایشات انجام و نتیجه پاسخ به درمان مشخص شد. پرسش‌نامه‌ای حاوی مشخصات دموگرافیک و علائم بیماری و عوارض دارویی تنظیم و از طریق مصاحبه با بیماران تکمیل شد. ملاک تعیین آلودگی، مشاهده حداقل یک انگل (تخم کرم یا کیست تروفوزوئیت تک‌یاخته در یک نمونه مدفوع) بود. نهایتاً نتایج تحلیل و به‌صورت آمار توصیفی ارائه شد. یافته‌ها: 64/7درصد از شرکت‌کنندگان مرد و 35/3درصد زن بودند. 124 نفر (93/2درصد) از مبتلایان به انگل‌های روده‌ای به درمان پاسخ داده وتنها 9 نفر (6/8درصد) به درمان پاسخ نداده بودند. اکثر افراد درگروه پاسخ به درمان زیر 30 سال (79/5درصد) و در گروه عدم پاسخ بالای 70 سال (26/7درصد) داشتند. میزان پاسخ به درمان در افراد آلوده به اکسیور 93/6درصد، انتامبا هیستولیتیکا / انتاموبا دیسپار 89/2درصد، ژیاردیا 90درصد، و دی انتاموبا فراژیلیس 100درصد بود. نتیجه‌گیری: با توجه به میزان بالای پاسخ به درمان، احتمال وجود مقاومت دارویی نسبت به داروهای ر ایج کم است و موارد عدم پاسخ به درمان می‌تواند در نتیجه مصرف ناصحیح دارو و یا شدت بالاتر آلودگی باشد.

Title: مقایسه کیفیت زندگی در سالمندان مبتلا به نارسایی مزمن کلیه با بیماران جوان و میانسال

http://salmandj.uswr.ac.ir/article-1-377-fa.html

اهداف: مطالعه حاضر با هدف مقایسه کیفیت زندگی سالمندان مبتلا به نارسایی مزمن کلیه با بیماران جوان و میانسال انجام شد. مواد و روش‌ها: در این مطالعه مقطعی، 202 بیمار مبتلا به نارسایی مزمن کلیه در سه گروه جوان (40≥)، میانسال (41 تا 59) و سالمند (60≤) قرار گرفتند. اطلاعات به روش سرشماری و با استفاده از پرسش‌نامه کیفیت زندگی فرنس و پوورس نسخه دیالیز جمع‌آوری شد. داده‌ها با به‌کارگیری نرم‌افزار spss و با استفاده از آزمون آنالیز واریانس و شفه تجزیه و تحلیل شد. یافته‌ها: 43/6درصد از شرکت‌کنندگان را سالمندان، 40/1درصد را افراد میانسال و 16/3درصد را جوانان تشکیل دادند. کیفیت زندگی کلی بیماران در سه گروه (سالمند، میانسال و جوان) تفاوت معنی‌دار آماری نداشت (0/05P> و 1/51F=). این در حالی است که بُعد اقتصادی و اجتماعی و خانوادگی کیفیت زندگی در سه گروه با یکدیگر تفاوت معنی‌دار آماری داشت (0/05P<). اما بُعد روانی‌معنوی و سلامتی و عملکرد در بین سه گروه تفاوت معنی‌دار آماری نداشت (0/05P>). نتیجه‌گیری: نتایج مطالعه حاکی از پایین‌بودن بُعد اقتصادی و اجتماعی و خانوادگی کیفیت زندگی جوانان نسبت به افراد سالمند و میانسال بود. بنابراین ارائه بیمه تکمیلی و ارجاع بیماران جوان به انجمن حمایت از بیماران پیوند و کلیه به منظور بهبود کیفیت زندگی این بیماران به خصوص در بُعد اقتصادی و اجتماعی پیشنهاد می‌شود.

Title: اثربخشی «درمان شناختی حضورذهن» بر شدت درد ادراک‌شده و محدودیت عملکرد زنان مبتلا به درد مزمن در روند سالمندی

http://salmandj.uswr.ac.ir/article-1-378-fa.html

اهداف: تعیین اثربخشی درمان شناختی حضورذهن کنترل درد، بر شدت درد ادراک‌شده و محدودیت عملکرد بیماران مبتلا به درد مزمن در کنار شناسایی رابطه این دو متغیر با سن، هدف پژوهش حاضر بود. مواد و روش‌ها: در این پژوهش نیمه تجربی، 30 بیمار زن داوطلب از بیماران 18 تا 65 ساله مبتلا به درد مزمن مراجعه‌کننده به بخش فیزیوتراپی بیمارستان اخوان به روش نمونه‌گیری در دسترس انتخاب شدند و در دو گروه مداخله و کنترل قرار گرفتند (15 نفر در هر گروه). برای جمع‌آوری اطلاعات مورد نیاز اعضای هر دو گروه با فاصله زمانی 2 ماه به پرسشنامه‌های طبقه‌بندی مدرج درد (GPQ) و ناتوانی ناشی از درد پاسخ دادند. برنامه 8  جلسه‌ای «درمان شناختی حضورذهن» در مورد گروه مداخله انجام شد و گروه کنترل هیچ مداخله روانشناختی دریافت نکرد. داده‌ها پس از جمع‌آ‌وری با استفاده از آزمون آماری تحلیل کوواریانس بررسی شدند. یافته‌ها: نتایج نشان داد در درجه اول سن با شدت درد ادراک‌شده و محدودیت عملکرد در بیماران مبتلا به درد مزمن رابطه دارد. همینطور نتایج تحلیل کوواریانس نشان داد که شدت درد ادراک‌شده (P=0/001) و محدودیت ناشی از درد (F=0/04) به طور معنی‌داری در گروه آزمایش نسبت به گروه کنترل کاهش یافت. نتیجه‌گیری: شدت درد ادراک‌شده و محدودیت عملکرد با روند افزایش سن رابطه دارد. درمان شناختی حضورذهن ممکن است با کاهش فاجعه‌آمیزکردن درد و در نتیجه کاهش اضطراب و ترس مرتبط با درد در کاهش شدت درد ادراک‌شده و محدودیت عملکرد بیماران مبتلا به درد مزمن مؤثر باشد و می‌تواند یک راهبرد پیشگیرانه مفیدی تلقی شود.
